# Supplementary material for: Notch signaling represses cone photoreceptor formation through the regulation of retinal progenitor cell states
Source: Sci Rep. 2021 Jul 15;11:14525. doi: 10.1038/s41598-021-93692-w (PMC8282820; doi:10.1038/s41598-021-93692-w)
Supplement: Supplementary file 1 — Supplementary Informations. [file 41598_2021_93692_MOESM1_ESM.pdf]

Supplementary Information File

Notch signaling represses cone photoreceptor formation through the regulation of retinal progenitor cell states

Xueqing Chen and Mark M. Emerson

Supplementary Fig. S1

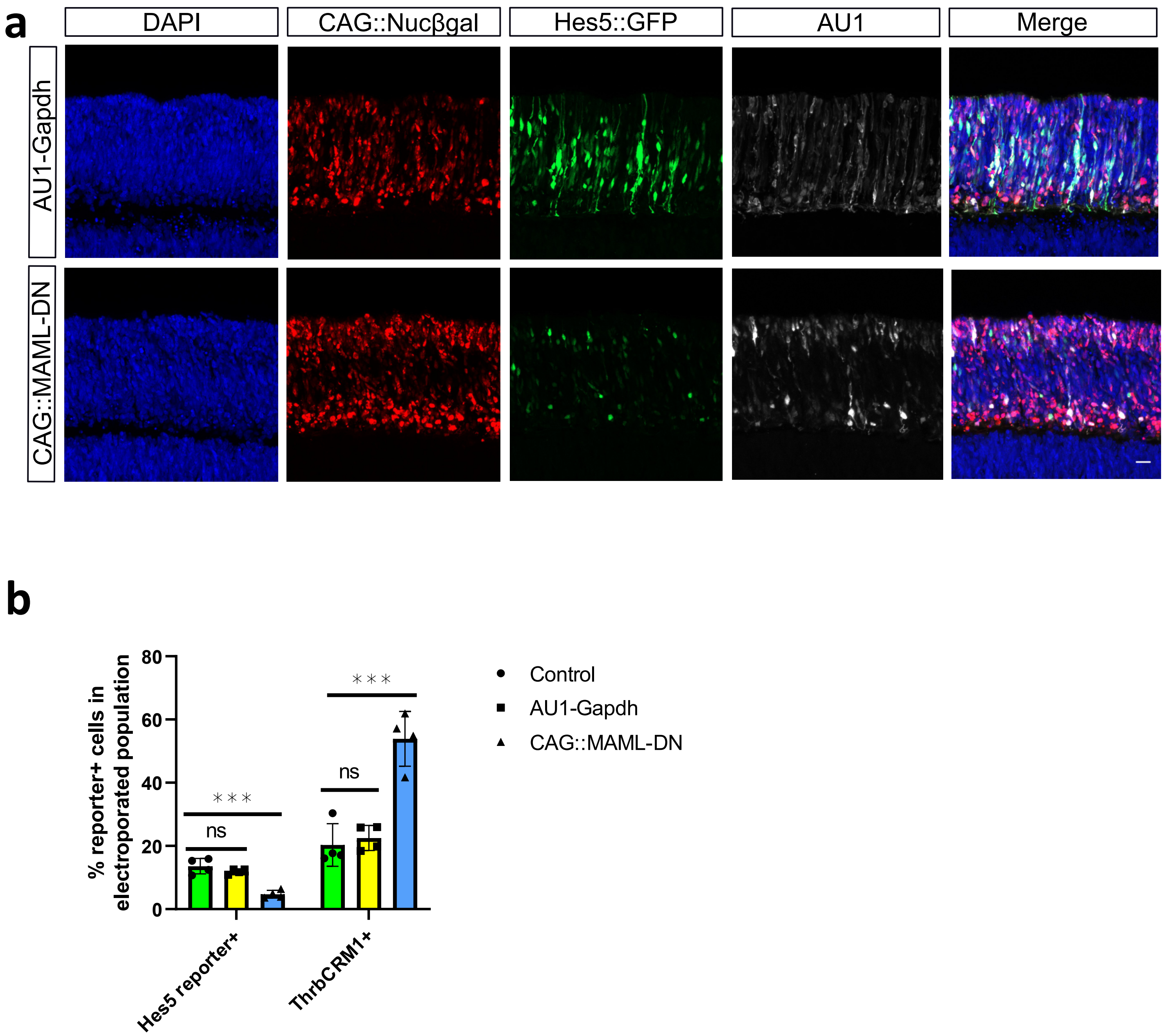

Supplementary Fig. S1. CAG::MAML-DN expression and validation in retinal cells

(a) Confocal images of vertically sectioned E5 chick retinas co-electroporated with CAG::Nucßgal, Hes5::GFP, and with CAG::MAML-DN or empty AU1-Gapdh vector and cultured for two days before AU1 immunostaining. The scale bar shown in the bottom right picture denotes 40 μm and applies to all images. All images are oriented with the scleral side of the retina at the top of the image. **(b)** Bar graphs of flow cytometry analysis of dissociated chick retinal cells electroporated ex vivo at E5 with the CAG::iRFP co-electroporation control, Hes5::GFP reporter, ThrbCRM1::TdT, and with CAG::MAML-DN or empty AU1-Gapdh vector and cultured for two days. The number of reporter-positive cells within the electroporated population is plotted. The Shapiro-Wilk normality test was used to confirm the normal distribution. *** signifies $p<0.001$ with a two-tailed student's t-test. Each point represents one biological replicate. The columns represent mean, and the error bars represent standard deviation.

Supplementary Fig. S2

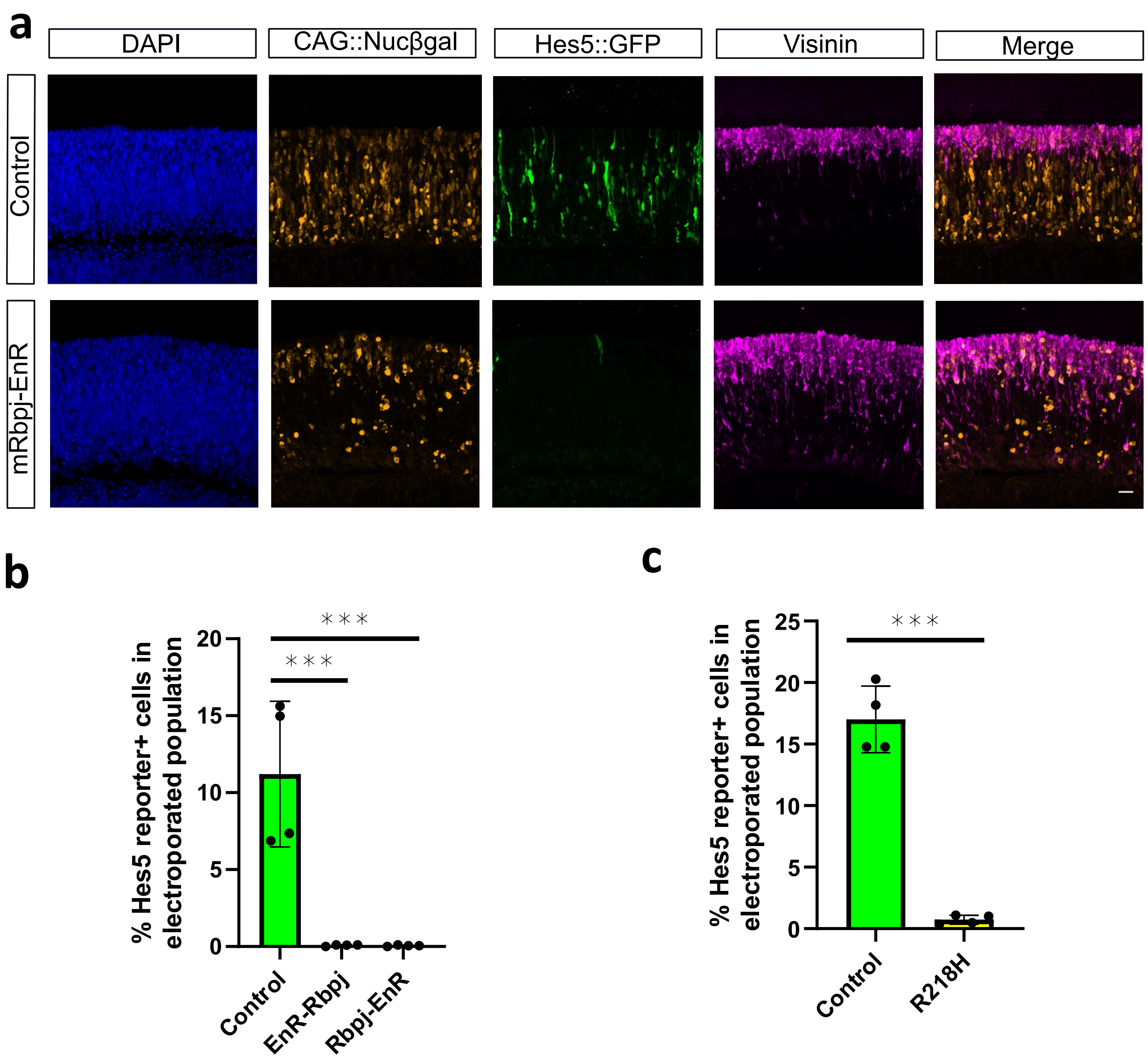

Supplementary Fig. S2. Rbpj-EnR and R218H have similar effects as CAG::MAML-DN on Hes5 Notch reporter

(a) Confocal images of vertically sectioned E5 chick retinas co-electroporated with CAG::Nucßgal, Hes5::GFP Notch reporter, and with or without Rbpj-EnR and cultured for two days before immunostained with Visinin. The scale bar shown in the bottom right picture denotes 40 μm and applies to all images. All images are oriented with the scleral side of the retina at the top of the image. **(b)** Flow cytometry quantification of the percentage of Hes5::GFP-positive cells within all the electroporated cells. Dissociated chick retinal cells electroporated ex vivo at E5 with the CAG::iRFP co-electroporation control, Hes5::GFP, and with or without EnR-Rbpj or Rbpj-EnR and cultured for two days. The Shapiro-Wilk normality test was used to confirm the normal distribution. ANOVA with a post hoc Dunn test was used to test significance. *** signifies $p<0.001$. Each point represents one biological replicate. The columns represent mean, and the error bars represent standard deviation. **(c)** Same as (a) but with or without R218H.

Supplementary Fig. S3

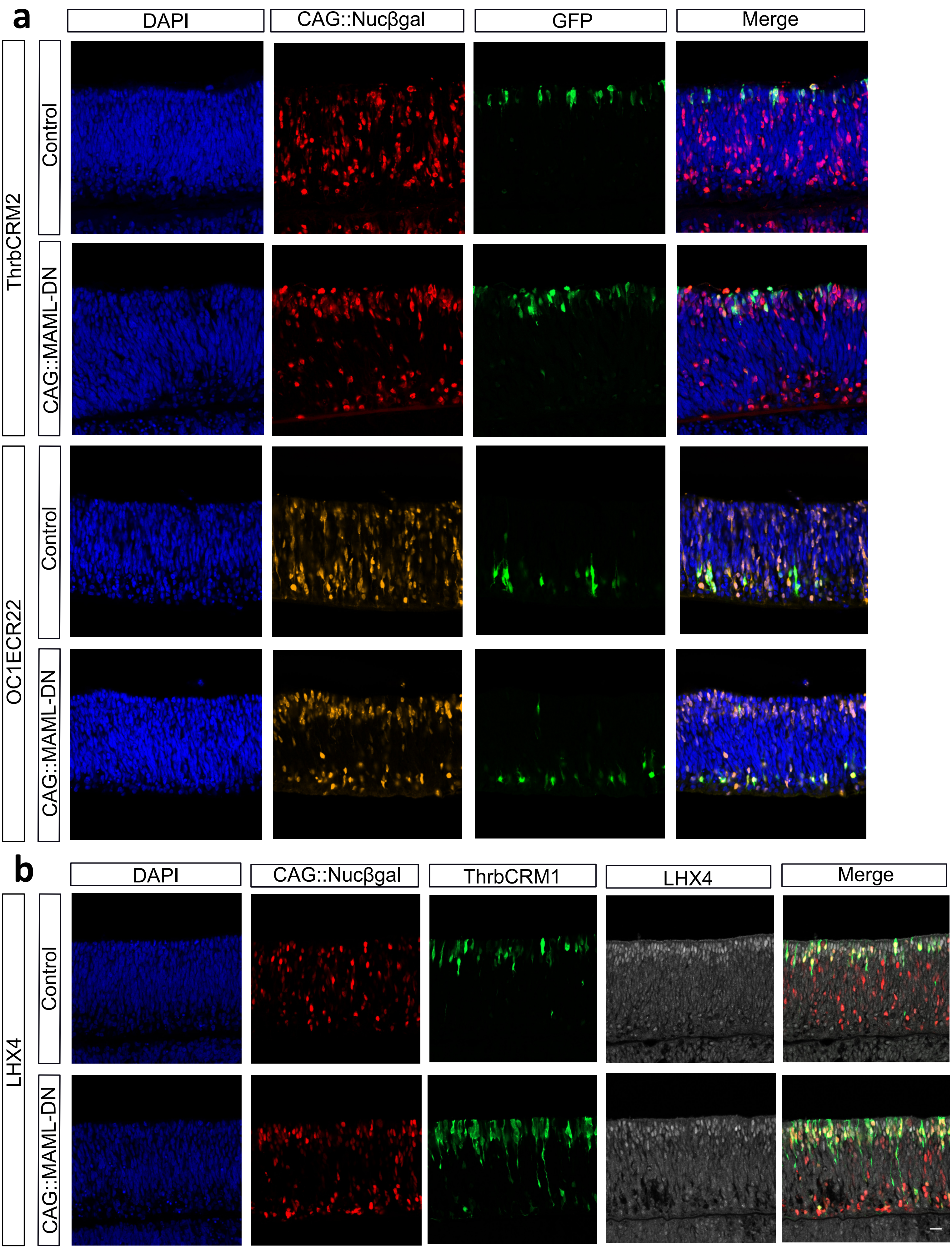

Supplementary Fig. S3. CAG::MAML-DN does not promote cone formation at two days post-electroporation

(a) Confocal images of vertically sectioned E5 chick retinas co-electroporated with CAG::Nucßgal, ThrbCRM2::GFP or OC1ECR22::GFP, and with or without CAG::MAML-DN and cultured for two days. The scale bar shown in the bottom right picture denotes 40 µm and applies to all images. All images are oriented with the scleral side of the retina at the top of the image. **(b)** Confocal images of vertically sectioned E5 chick retinas co-electroporated with CAG::Nucßgal, ThrbCRM1::GFP , and with or without CAG::MAML-DN and cultured for two days. LHX4 antibody was used to label cones. The scale bar shown in the bottom right picture denotes 40 µm and applies to all images. All images are oriented with the scleral side of the retina at the top of the image.

Supplementary Fig. S4

a

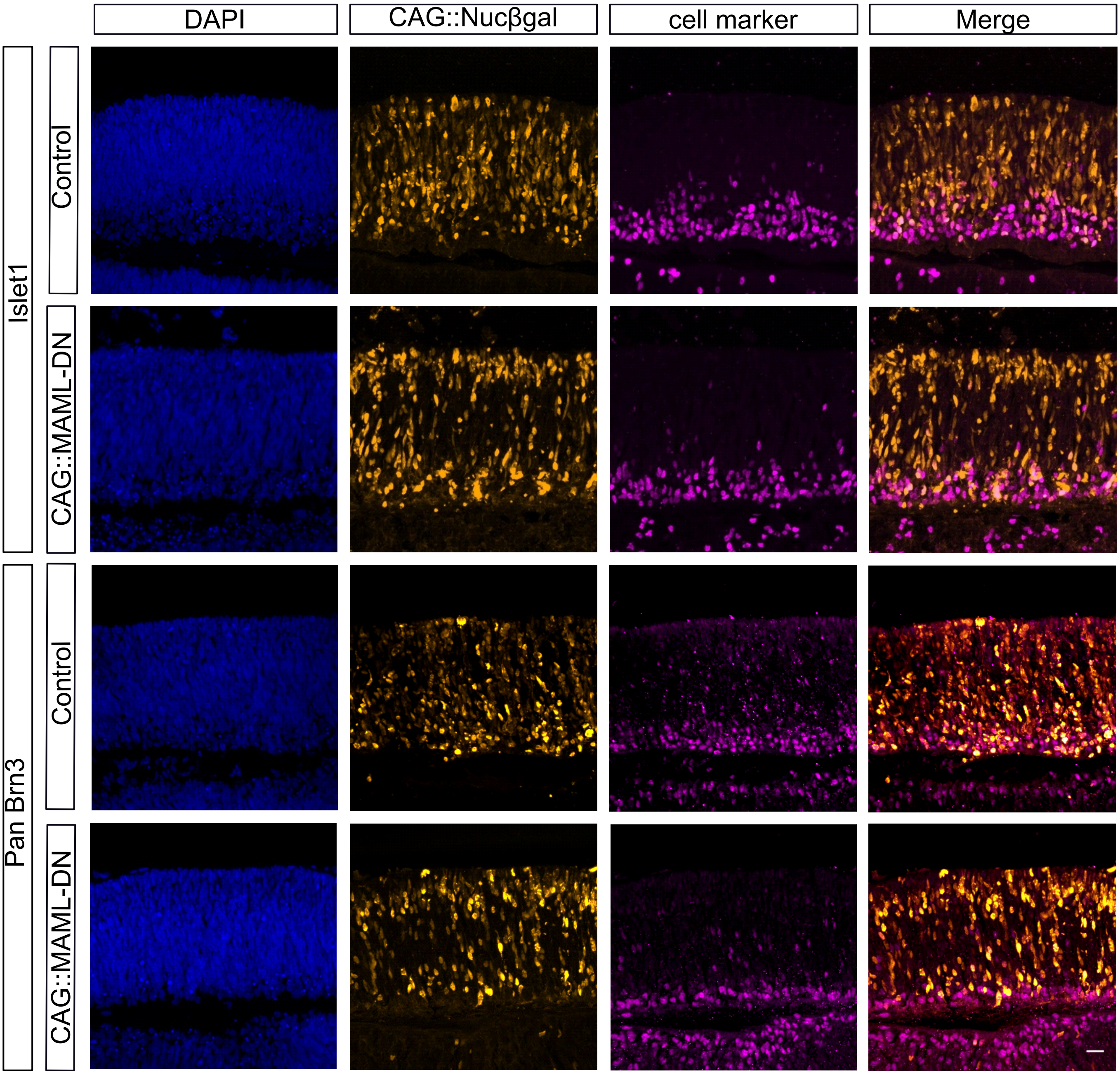

b

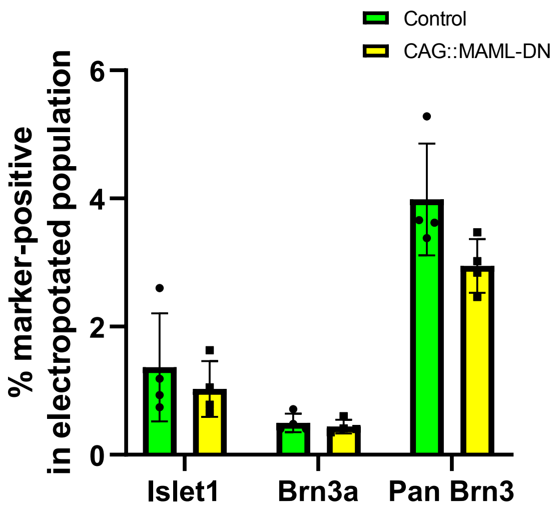

Supplementary Fig. S4. CAG::MAML-DN does not shift the number of H2, H3 and H4 HCs and RGCs

(a) Confocal images of vertically sectioned E5 chick retinas co-electroporated with CAG::Nucßgal, and with or without CAG::MAML-DN and cultured for two days. Sections were immunostained with Islet1 and Pan Brn3 cell specific markers (magenta), CAG::Nucßgal (orange), and nuclei visualized with DAPI. The scale bar in the bottom right panel denotes 40 μm and applies to all images. All images are oriented with the scleral side of the retina at the top of the image. **(b)** Flow cytometry quantification of the percentage of Islet1, Brn3a and Pan Brn3-positive cells within all electroporated cells. Dissociated chick retinal cells electroporated *ex vivo* at E5 with the CAG::TdTomato co-electroporation control and cultured for two days. The Shapiro-Wilk normality test was used to confirm the normal distribution. A two-tailed student's t-test was used to test significance in Islet1 and Brn3a quantifications. Mann-Whitney test was used to test significance in Pan Brn3 quantification. Each point represents one biological replicate. The columns represent mean, and the error bars represent standard deviation.

Supplementary Fig. S5

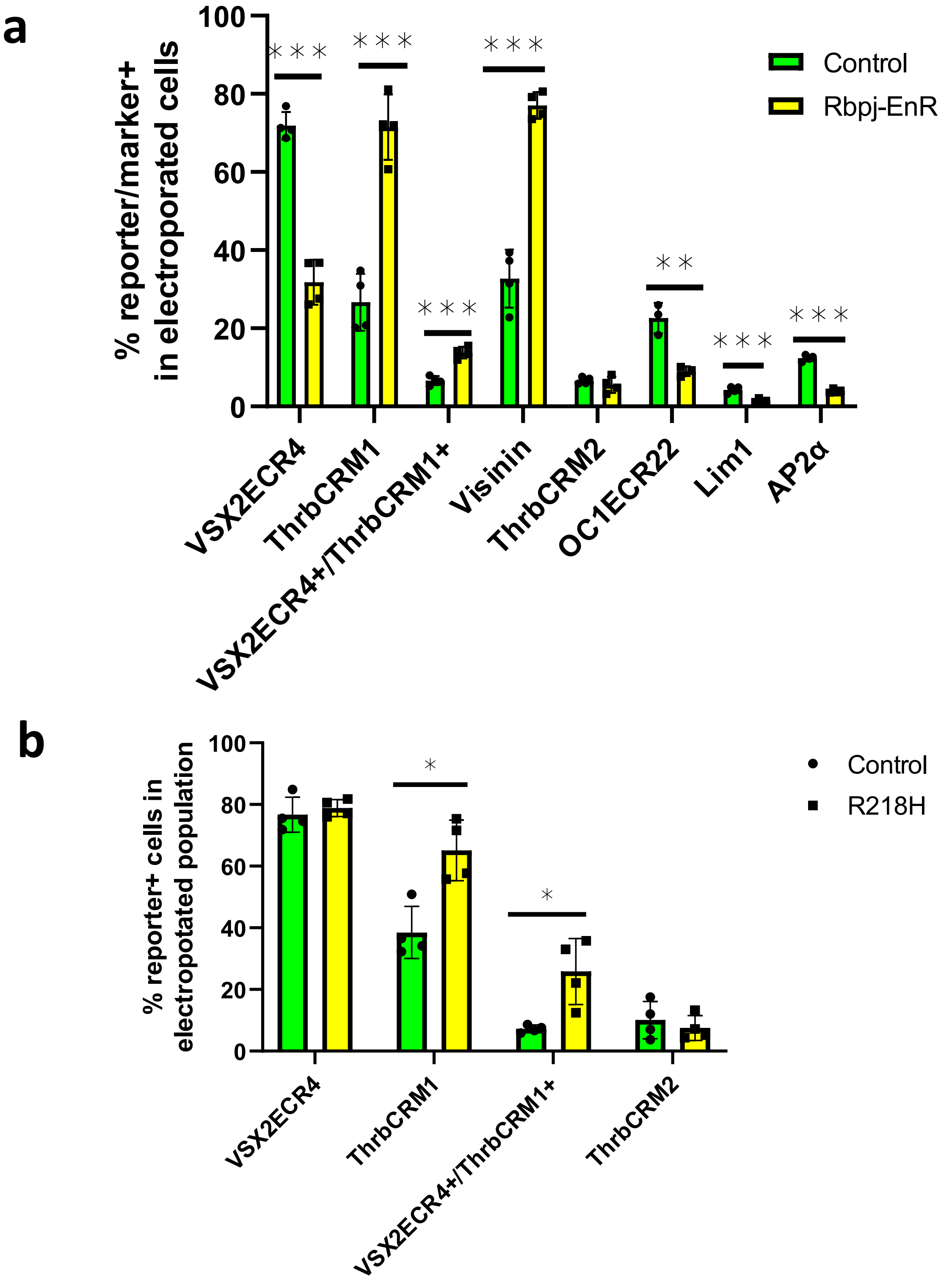

Supplementary Fig. S5. Rbpj-EnR and R218H have similar effects as CAG::MAML-DN on ThrbCRM1 RPCs

(a) Flow cytometry quantification of the percentage of VSX2ECR4::GFP, ThrbCRM1::TdTomato, VSX2ECR4::GFP/ThrbCRM1::TdTomato double reporter-positive, Visinin, ThrbCRM2::TdTomato, OC-ECR22::GFP, Lim1 and AP2α-positive cells within all the electroporated cells. Dissociated chick retinal cells electroporated ex vivo at E5 with the CAG::iRFP or CAG::TdTomato co-electroporation control, and with or without Rbpj-EnR and cultured for two days. The Shapiro-Wilk normality test was used to confirm the normal distribution. ** signifies $p<0.01$, *** signifies $p<0.001$ with a two-tailed student's t-test. **(b)** Flow cytometry quantification of the percentage of ThrbCRM2::TdTomato, VSX2ECR4::GFP, ThrbCRM1::TdTomato, VSX2ECR4::GFP/ThrbCRM1::TdTomato double reporter-positive cells within all the electroporated cells. Dissociated chick retinal cells electroporated ex vivo at E5 with the CAG::iRFP co-electroporation control, and with or without R218H and cultured for two days. The Shapiro-Wilk normality test was used to confirm the normal distribution. ANOVA with a post hoc Dunn test was used to test significance. * signifies $p<0.05$. Each point represents one biological replicate. The columns represent mean, and the error bars represent standard deviation.

Supplementary Fig. S6

a

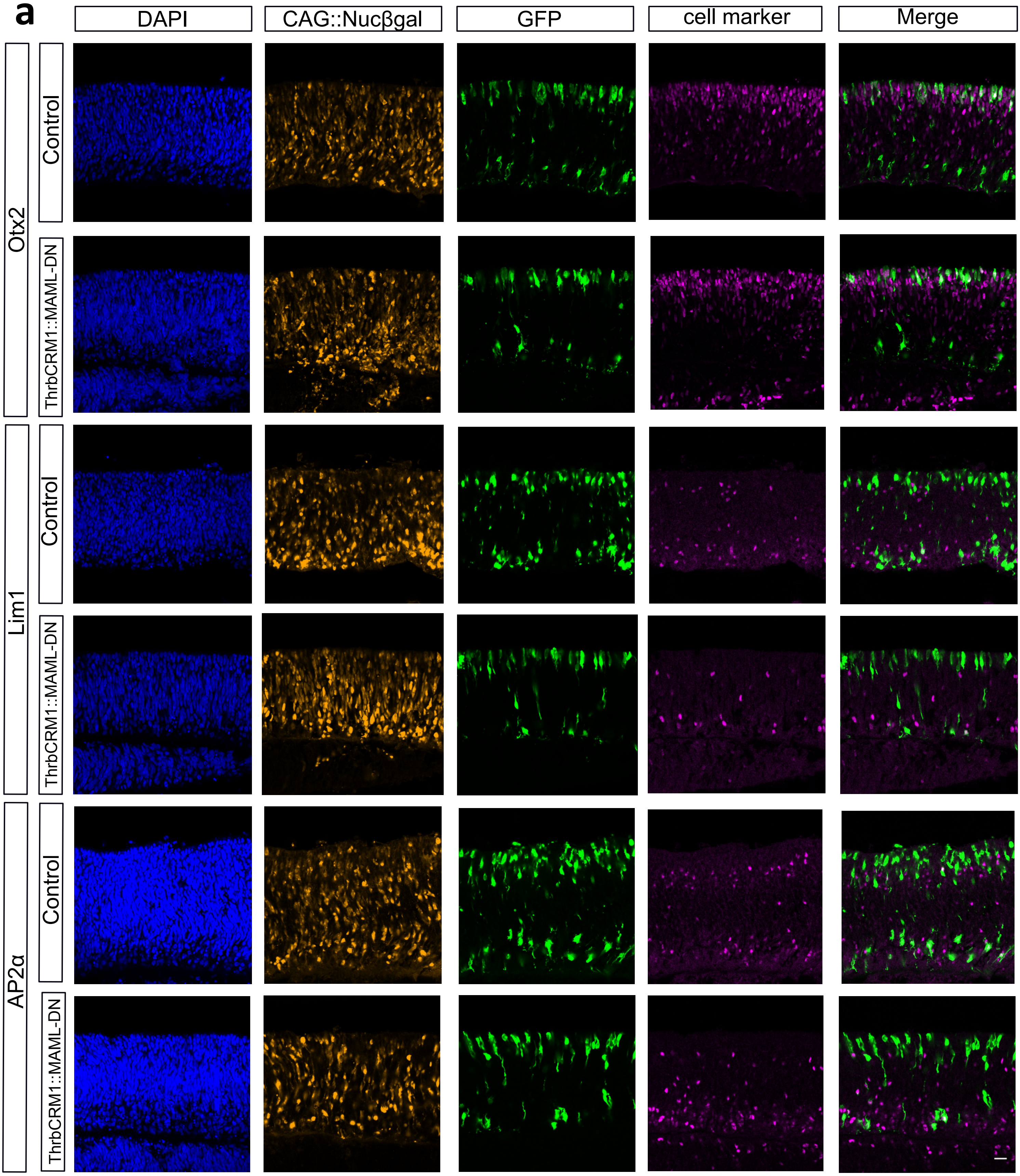

b

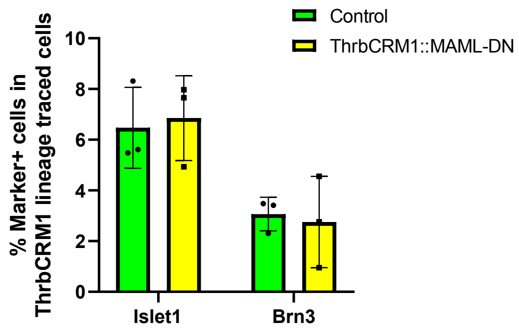

Supplementary Fig. S6. ThrbCRM1::MAML-DN does not have effects on H2, H3 and H4 HCs and RGCs within ThrbCRM1 lineage traced population

(a) Confocal images of vertically sectioned E5 chick retinas co-electroporated with CAG::Nucßgal, ThrbCRM1::PhiC31 and its responder plasmid, and with or without ThrbCRM1::MAML-DN. The retinas were cultured for two days post-electroporation. The sections were immunostained with Otx2, Lim1 and AP2α markers (magenta), CAG::Nucßgal (orange), and nuclei visualized with DAPI. The scale bar shown in the bottom right picture denotes 40 μm and applies to all images. All images are oriented with the scleral side of the retina at the top of the image. **(b)** Quantification of the percentage of Islet1 and Brn3 marker-positive cells within the ThrbCRM1 lineage traced cell population from cell counting. Sectioned chick retinas electroporated *ex vivo* at E5 with the co-electroporation control CAG::Nucßgal, ThrbCRM1::PhiC31 and its responder plasmid, and with or without ThrbCRM1::MAML-DN. The retinas were cultured for two days after electroporation. The Shapiro-Wilk normality test was used to confirm the normal distribution. A two-tailed student's t-test was used to test significance. Each point represents one biological replicate. The columns represent mean, and the error bars represent standard deviation.

Supplementary Fig. S7

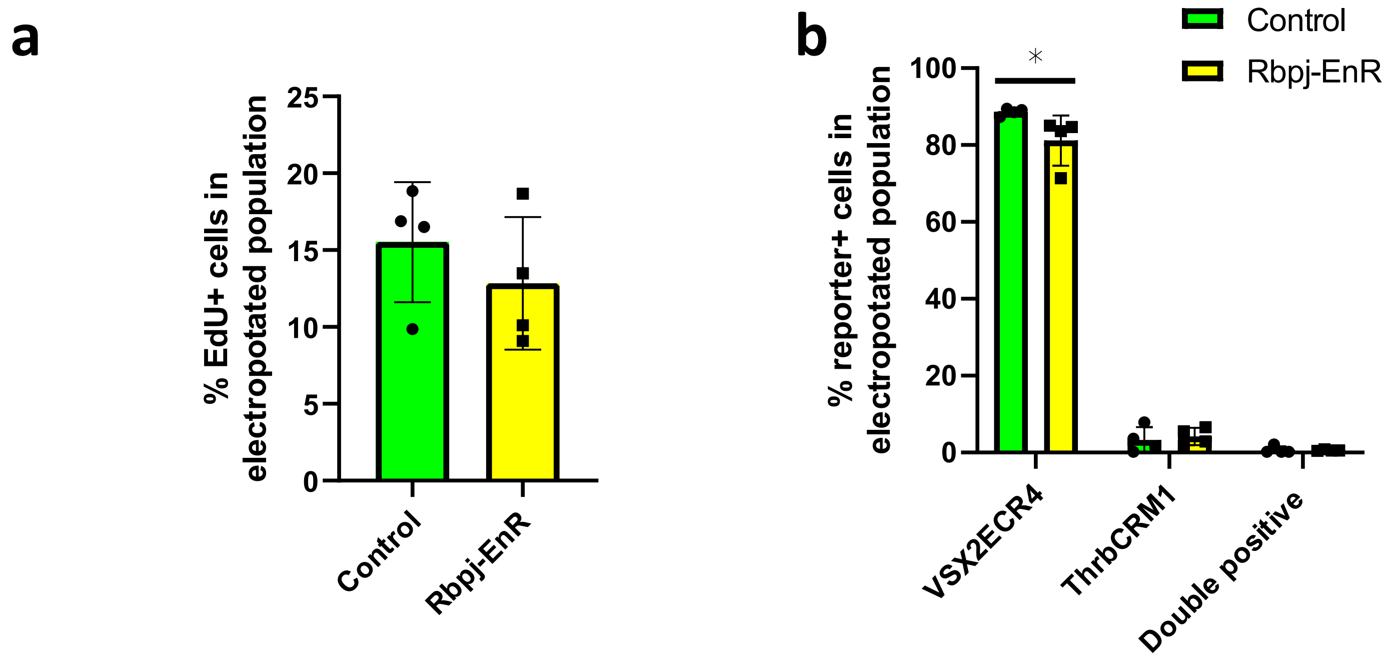

Supplementary Fig. S7. Rbpj-EnR induced Notch inhibition has similar effects as CAG::MAML-DN on cell proliferation

(a) Flow cytometry quantification of the percentage of EdU-positive cells within all the electroporated cells. Dissociated chick retinal cells electroporated ex vivo at E5 with the co-electroporation control CAG::TdTomato with or without Rbpj-EnR. The retinas were cultured for two days after electroporation and pulsed with EdU for 1 hour before harvest. The Shapiro-Wilk normality test was used to confirm the normal distribution. A two-tailed student's t-test was used to test significance. **(b)** Flow cytometry quantification of the percentage of VSX2ECR4, ThrbCRM1 or VSX2ECR4/ ThrbCRM1 reporter-positive cells within the electroporated cell population. Dissociated chick retinal cells electroporated ex vivo at E5 with the co-electroporation control CAG::iRFP, VSX2ECR4::GFP or ThrbCRM1::TdTomato, and with or without Rbpj-EnR. The retinas were cultured for 8 hours post-electroporation. The Shapiro-Wilk normality test was used to confirm the normal distribution. A two-tailed student's t-test was used to test significance in ThrbCRM1 quantification. Mann-Whitney test was used to test significance in VSX2ECR4 and double reporter-positive quantifications. * signifies $p<0.05$. Each point represents one biological replicate. The columns represent mean, and the error bars represent standard deviation.

Supplemental Table 1

Graphs	Groups	Replicate	Control mean(%)	Control SD(%)	Dominant-negative mean(%)	Dominant-negative SD(%)	P-value
Fig 1C	Hes5::GFP	1	9.76	2.27	2.60	2.00	0.0011
		2	12.43	3.07	5.03	2.13	0.0075
Fig 2B	VSX2ECR4	1	59.54	5.79	45.51	5.47	0.0124
		2	70.14	3.43	56.67	8.57	0.0266
	ThrbCRM1	1	19.93	3.57	44.08	4.04	0.0001
		2	34.76	5.08	66.25	10.79	0.0019
	ThrbCRM2	1	3.16	1.18	4.64	1.63	0.1924
		2	4.46	1.79	5.82	4.10	0.5658
	OC1ECR22	1	8.00	1.09	8.15	0.67	0.8197
		2	7.68	2.32	7.33	2.25	0.8350
	VSX2ECR4 ThrbCRM1	1	4.91	1.42	23.96	3.78	0.0001
	double positive	2	2.12	0.35	11.76	6.96	0.0230
Fig 3B	Visinin	1	25.36	3.28	57.50	5.31	0.0001
		2	18.44	5.45	31.29	8.25	0.0408
	Otx2	1	42.58	1.84	60.13	3.78	0.0002
		2	27.37	1.69	50.90	4.91	0.0001
	Lim1	1	9.48	0.84	5.73	1.61	0.0062
		2	9.48	1.07	6.09	1.54	0.0111
	AP2α	1	5.42	0.20	4.39	0.37	0.0011
		2	9.37	2.67	5.73	0.69	0.0383
Fig 4D	Visinin	1	74.00	3.81	74.41	3.54	0.8784
		2	81.77	8.56	89.56	5.79	0.1823
	Otx2	1	79.85	3.02	78.42	4.38	0.6112
		2	59.5	9.97	58.99	3.37	0.9259
	Lim1	1	17.43	4.06	11.15	0.68	0.0225
		2	11.22	1.25	8.89	0.52	0.0135
	AP2α	1	15.97	1.23	11.86	0.53	0.0010
		2	18.99	2.13	12.11	0.37	0.0090
	OC1ECR22	1	17.03	1.79	10.31	2.09	0.0028
		2	11.98	1.08	9.74	0.38	0.0079
	ThrbCRM2	1	9.42	2.19	16.26	5.63	0.1143
		2	5.46	4.68	5.16	2.8	0.9184
Fig 5A	EdU	1	11.04	2.99	8.69	2.04	0.2411
		2	17.33	3.89	15.28	1.74	0.3731
Fig 5E	VSX2ECR4 ThrbCRM1	1	4.90	1.78	21.51	7.61	0.0051
	double positive	2	3.37	0.29	16.49	6.76	0.0082
Fig 6B	VSX2ECR4	1	89.54	1.84	88.08	2.75	0.8857
		2	88.60	0.96	84.95	4.02	0.1280
	ThrbCRM1	1	4.71	1.89	3.63	1.62	0.4183
		2	3.28	3.37	5.41	1.91	0.3145
	Double positive	1	1.47	0.69	0.73	0.24	0.0890
		2	0.68	0.95	1.4	0.83	0.2984
Fig 7B	VSX2ECR4+ 2d	1	60.55	13.83	58.11	17.61	0.8347
		2	60.06	8.52	61.67	9.04	0.8035
	ThrbCRM2+ 2d	1	3.16	1.18	4.64	1.63	0.1924
		2	11.13	2.64	9.75	3.31	0.5364
	Double positive 2d	1	0.54	0.14	1.38	1.17	0.2015
		2	1.48	0.41	1.81	0.69	0.4349
	VSX2ECR4+ 3d	1	61.58	11.93	53.12	4.90	0.2371
		2	73.19	7.12	64.9	0.93	0.0605
	ThrbCRM2+ 3d	1	11.71	7.47	13.26	2.01	0.7024
		2	20.98	3.86	21.77	2.82	0.7523
	Double positive 3d	1	1.88	0.58	3.35	0.03	0.0023
		2	3.75	1.02	5.98	2.89	0.0339
Fig 7C	VSX2ECR4+ 4d	1	72.91	8.36	59.61	11.57	0.1117
		2	41.70	7.88	33.49	13.86	0.3425
	ThrbCRM2+ 4d	1	23.66	16.06	61.00	8.23	0.0061

		**2**	15.26	4.89	33.62	3.5	0.0009
	**Double positive 4d**	**1**	3.89	1.70	24.92	6.11	0.0006
		**2**	3.16	1.37	15.89	3.88	0.0008
	**VSX2ECR4+ 5d**	**1**	78.12	8.64	72.52	19.23	0.6138
		**2**	84.87	8.17	72.69	6.86	0.0624
	**ThrbCRM2+ 5d**	**1**	19.13	12.92	42.58	6.91	0.0186
		**2**	13.04	2.95	34.75	6.7	0.0010
	**Double positive 5d**	**1**	2.37	1.19	9.35	3.12	0.0058
		**2**	2.19	0.63	12.07	5.44	0.0113
**Fig S5A**	**ThrbCRM1**	**1**	26.69	7.31	71.49	8.37	0.0002
		**2**	21.51	1.41	60.34	2.9	0.0001
	**ThrbCRM2**	**1**	6.62	0.87	5.54	2.06	0.3707
		**2**	8.39	2.41	5.94	0.96	0.1771
	**OC1ECR22**	**1**	22.61	3.93	9.02	1.26	0.0047
		**2**	19.02	1.57	15.21	2.04	0.0254
**Fig S5B**	**ThrbCRM1**	**1**	38.48	8.45	65.12	9.82	0.0485
		**2**	21.01	1.7	59.32	4.04	0.0286
**Fig S7A**	**EdU**	**1**	15.53	3.91	12.85	4.31	0.3928
		**2**	20.14	4.09	16.09	1.6	0.1153

			**Control**		**Dominant-negative1**			**Dominant-negative2**		
**Graphs**	**Groups**	**Replicate**	**mean(%)**	**SD(%)**	**mean(%)**	**SD(%)**	**P value1**	**mean(%)**	**SD(%)**	**P value2**
**Fig 4A**	**ThrbCRM1**	**1**	16.81	4.81	30.56	5.74	0.0011	17.37	0.30	0.3093
		**2**	14.10	1.82	25.21	3.37	0.0001	13.19	1.07	0.1532
